# Antinociceptive Effects of Cardamonin in Mice: Possible Involvement of TRPV_1_, Glutamate, and Opioid Receptors

**DOI:** 10.3390/molecules23092237

**Published:** 2018-09-03

**Authors:** Chung Pui Ping, Tengku Azam Shah Tengku Mohamad, Muhammad Nadeem Akhtar, Enoch Kumar Perimal, Ahmad Akira, Daud Ahmad Israf Ali, Mohd Roslan Sulaiman

**Affiliations:** 1 Department of Biomedical Sciences, Faculty of Medicine and Health Sciences, Universiti Putra Malaysia Selangor, Serdang 43400, Malaysia; puiping.chung@gmail.com (C.P.P.); azamshah@upm.edu.my (T.A.S.T.M.); enoch@upm.edu.my (E.K.P.); ahmadakira@upm.edu.my (A.A.); daudaia@upm.edu.my (D.A.I.A.); 2 Laboratory of Natural Products, Institute of Bioscience, Universiti Putra Malaysia, Selangor, Serdang 43400, Malaysia; nadeemupm@gmail.com; 3 Faculty of Industrial Sciences & Technology, University Malaysia Pahang, Pahang, Gambang 26300, Malaysia

**Keywords:** cardamonin, antinociceptive, TRPV_1_, glutamate, opioid

## Abstract

Pain is one of the most common cause for hospital visits. It plays an important role in inflammation and serves as a warning sign to avoid further injury. Analgesics are used to manage pain and provide comfort to patients. However, prolonged usage of pain treatments like opioids and NSAIDs are accompanied with undesirable side effects. Therefore, research to identify novel compounds that produce analgesia with lesser side effects are necessary. The present study investigated the antinociceptive potentials of a natural compound, cardamonin, isolated from *Boesenbergia rotunda* (L) Mansf. using chemical and thermal models of nociception. Our findings showed that intraperitoneal and oral administration of cardamonin (0.3, 1, 3, and 10 mg/kg) produced significant and dose-dependent inhibition of pain in abdominal writhing responses induced by acetic acid. The present study also demonstrated that cardamonin produced significant analgesia in formalin-, capsaicin-, and glutamate-induced paw licking tests. In the thermal-induced nociception model, cardamonin exhibited significant increase in response latency time of animals subjected to hot-plate thermal stimuli. The rota-rod assessment confirmed that the antinociceptive activities elicited by cardamonin was not related to muscle relaxant or sedative effects of the compound. In conclusion, the present findings showed that cardamonin exerted significant peripheral and central antinociception through chemical- and thermal-induced nociception in mice through the involvement of TRPV_1_, glutamate, and opioid receptors.

## 1. Introduction

Cardamonin or 2′,4′-dihydroxy-6′-methoxychalcone (C_16_H_14_O_4_) is a naturally occurring chalcone. Cardamonin was firstly isolated from the seeds of *Amomum subulatum* [1] and later from other plant species, such as *Boesenbergia pandurata*, *Alpinia rafflesiana*, *Alpinia katsumadai*, *Alpinia henryi*, and *Campomanesia adamantium*. Previous report showed that cardamonin exerted antiproliferative activity and induced apoptosis in PC-3 [2], myeloma [3], and A549 cell lines [4]. In addition, cardamonin was capable to protect lipopolysaccharide (LPS)-induced septic mice against acute lung injury [5] and showed nephroprotective effect against cisplatin-induced renal injury [6]. Furthermore, cardamonin could exert inhibition of platelet aggregation [7], vasorelaxant effect [8], improvement in insulin resistance and vascular complication in a high fructose-fed rat model [9], suppression of lipid accumulation in vitro [10], inhibition of pigmentation in human normal melanocytes [11], and anti-pruritic activity [12].

In vitro study showed that cardamonin inhibits the release of pro-inflammatory mediators [13] and it was supported by the findings on inhibitory action of cardamonin upon the expression of NO and PGE_2_ via interruption of the NF-κB pathway [14,15]. This mechanism of action is common to phenolic compounds showing protective effects [16,17]. In vivo study also presented the capability of cardamonin in suppressing NO generation in LPS-challenged ICR mice [18]. NO has been reported to be involved in generation of pain perception through a series of pathway [19]. Since cardamonin reduces generation of NO, we believe that cardamonin may show antinociceptive activityies.

Recently, the antinociceptive profile of cardamonin has been reported through PBQ-induced writhing and carrageenan-induced hyperalgesia test [20], but there is still no report on the possible mechanism of antinociceptive action of cardamonin. Thus, the present study was aimed to examine the antinociceptive effect of cardamonin in chemical- and thermal-induced nociception using mice models.

## 2. Results

### 2.1. Evaluation of the Antinociceptive Activity

#### 2.1.1. Acetic Acid–Induced Abdominal Writhing Test

**Figure 1:**
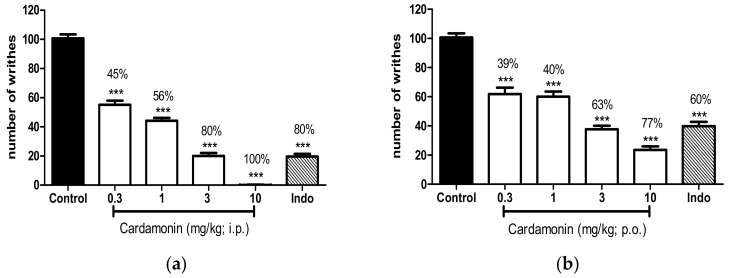
(**a**) Effect of cardamonin (0.3, 1, 3, 10 mg/kg, i.p.) administered intraperitoneally against acetic acid-induced nociception. (**b**) Effect of cardamonin (0.3, 1, 3, 10 mg/kg, i.p.) administered orally against acetic acid-induced nociception. Each column represents the mean ± S.E.M. of 6 mice. Control group received only the vehicle (ethanol: Tween 20: distilled water in 5:5:90, *v*/*v*/*v*) used to dilute the compound. Indomethacin (Indo, 10 mg/kg) was used as positive control. The asterisks denote the significance levels compared with the control group (one-way ANOVA, followed by Dunnett’s post hoc test); *** *p* < 0.001. Values in parentheses were percentage of inhibition.

The effect of cardamonin in acetic acid-induced abdominal writhing response in mice is depicted in Figure 1. Cardamonin produced significant reduction in the number of writhing in both routes of administration, intraperitoneally (Panel a) and orally (Panel b) at *p* < 0.001. Cardamonin administered intraperitoneally (i.p.) showed 45, 56, 80, and 100% of inhibition against acetic acid-induced pain as compared to control at doses of 0.3, 1, 3, and 10 mg/kg respectively, with calculated ED_50_ (and its respective confidence interval) of 2.1 (1.9–2.5) mg/kg. Oral administration (p.o.) of cardamonin exhibited 39%, 40%, 63% and 77% of inhibition as compared to control at doses of 0.3, 1, 3, and 10 mg/kg, respectively, with calculated ED_50_ (and its respective confidence interval) of 2.5 (2.0–3.3) mg/kg. Indomethacin, produced significant inhibition in acetic acid-induced abdominal pain with 80% (i.p.) and 60% (p.o.) of inhibition respectively, as compared to control at *p* < 0.001.

#### 2.1.2. Formalin-Induced Paw Licking Test

**Figure 2:**
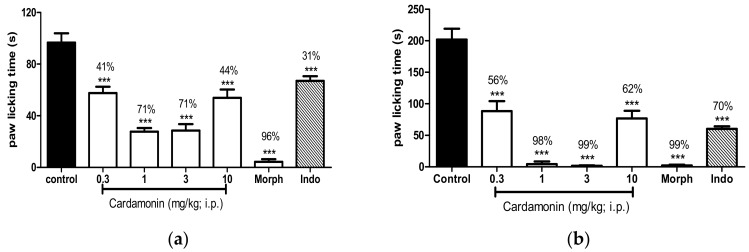
(**a**) Effect of cardamonin (0.3, 1, 3, 10 mg/kg, i.p.) in formalin-induced paw licking test (early phase) in mice. (**b**) Effect of cardamonin (0.3, 1, 3, 10 mg/kg, i.p.) in formalin-induced paw licking test (late phase) in mice. Each column represents the mean ± S.E.M. of 6 mice. Control group received only the vehicle used to dilute the compound. Morphine (Morph, 5 mg/kg, s.c.) and indomethacin (Indo, 10 mg/kg, i.p.) were used as positive control. The asterisks denote the significance levels compared with the control group (one-way ANOVA, followed by Dunnett’s post hoc test); *** *p* < 0.001. Values in parentheses were percentage of inhibition.

Cardamonin treated animals showed significant analgesic effect on both early neurogenic phase (0–5 min) and late inflammatory phase (15–35 min) in the formalin-induced paw licking test as shown in Figure 2 (Panel a and b respectively). Cardamonin inhibited the inflammatory pain better in comparison with the neurogenic pain at all dosage use, especially at 1 mg/kg (98%) and 3 mg/kg (99%) against inflammatory pain. Morphine (5 mg/kg; s.c.) produced significant inhibition (*p* < 0.001) against formalin-induced pain at both the early neurogenic phase (96%) and late inflammatory phase (99%). In contrast, indomethacin significantly inhibits formalin-induced pain better at the late inflammatory phase (70% of inhibition) than the early neurogenic phase (31% of inhibition).

#### 2.1.3. Hot Plate Test

i.p. administration of cardamonin and morphine significantly increased the latency time of nociceptive responses in the hot plate test at the temperature of 52 ± 0.2 °C compared with vehicle-treated animals (Table 1).

### 2.2. Investigation of the Mechanisms of Action

#### 2.2.1. Involvement of the TRPV_1_ Receptor

**Figure 3:**
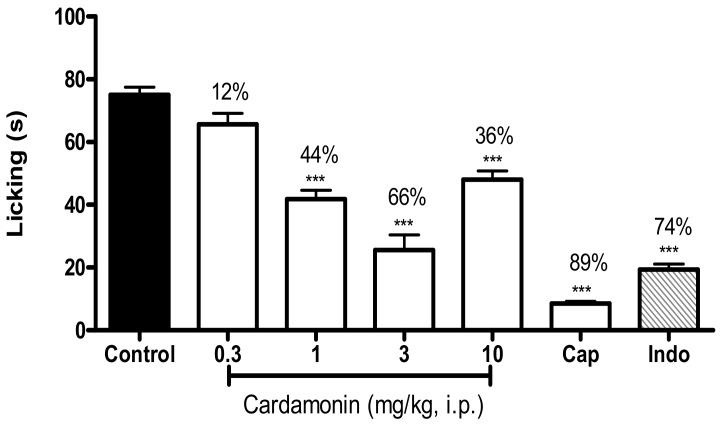
Effect of cardamonin (0.3, 1, 3, 10 mg/kg, i.p.) against capsaicin-induced nociception. Each column represents the mean ± S.E.M. of 6 mice. Control group receives only the vehicle used to dilute the compound. Capsazepine (Cap, 0.17 mmol/kg, i.p.) was used as the positive control for capsaicin-induced nociception. Indomethacin (Indo, 10 mg/kg, i.p.) was used as positive control. The asterisks denote the significance levels compared with the control group (one-way ANOVA, followed by Dunnett’s post hoc test); *** *p* < 0.001. Values in parentheses are percentage of inhibition.

Cardamonin showed significant inhibition against capsaicin-induced nociception at 1, 3 and 10 mg/kg with *p* < 0.001 in Figure 3. The maximum inhibition was produced by cardamonin at the dose of 3 mg/kg with 66% inhibition compared to control. Likewise, capsazepine and indomethacin significantly exhibited 89% and 74% inhibition, respectively.

#### 2.2.2. Involvement of the Glutamate Receptor

**Figure 4:**
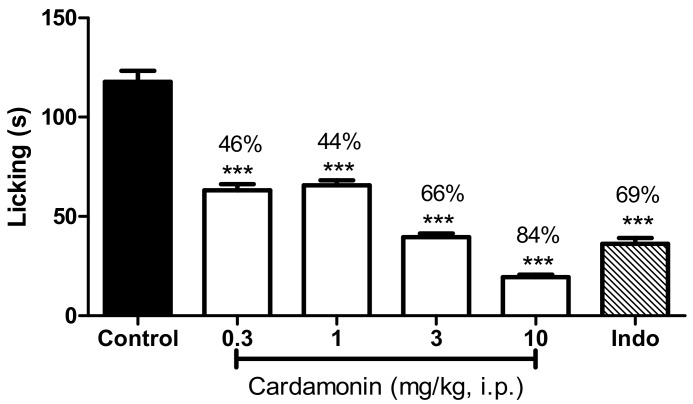
Effect of cardamonin (0.3, 1, 3, 10 mg/kg, i.p.) against glutamate-induced nociception. Each column represents the mean ± S.E.M. of 6 mice. Control group receives only the vehicle used to dilute the compound. Indomethacin (Indo, 10 mg/kg, i.p.) was used as positive control. The asterisks denote the significance levels compared with the control group (one-way ANOVA, followed by Dunnett’s post hoc test), *** *p* < 0.001. Values in parentheses are percentage of inhibition.

Using the glutamate-induced nociception study, it was observed that cardamonin produced significant antinociceptive activities at all dosage (Figure 4). The percentage of inhibition for were 46%, 44%, 66%, and 84% for 0.3, 1, 3, and 10 mg/kg of cardamonin, respectively, as in comparison with control. Indomethacin showed 69% of significant inhibition against glutamate-induced nociception at *p* < 0.001.

#### 2.2.3. Involvement of the Opioid Receptors

**Figure 5:**
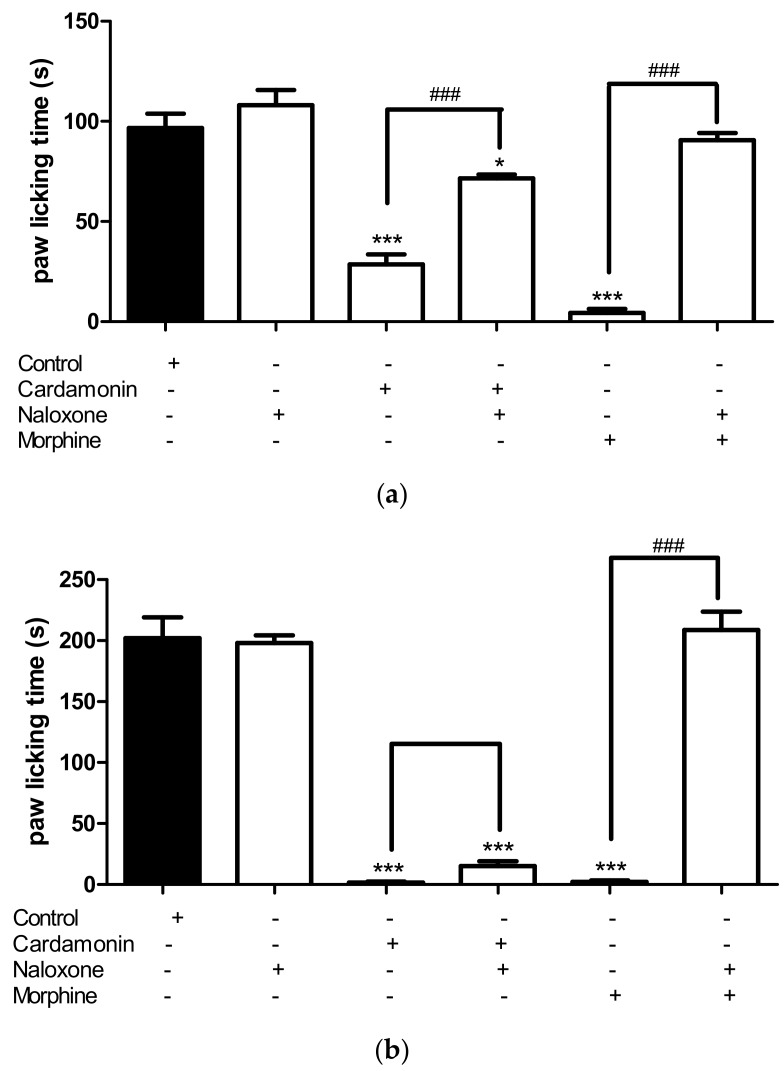
(**a**) Effect of naloxone (5 mg/kg, i.p.) on antinociception caused by cardamonin (1 mg/kg, i.p.) in the early phase in formalin-induced paw licking test. (**b**) Effect of naloxone (5 mg/kg, i.p.) on antinociception caused by cardamonin (1 mg/kg, i.p.) in the and the late phase in formalin-induced paw licking test. The asterisks and hash denote the significance levels (one-way ANOVA, followed by Tukey’s post hoc test), * *p* < 0.05, *** *p* < 0.001, ^###^ *p* < 0.001.

Pre-treatment of mice with the non-specific opioid receptor antagonist, naloxone (5 mg/kg; i.p.) significantly reversed the antinociceptive effect of cardamonin (1 mg/kg; i.p.) at *p* < 0.001 only in the early neurogenic phase, but not the late inflammatory phase (Figure 5). The effect of morphine (5 mg/kg; s.c.) was significantly reversed by naloxone in both the early neurogenic phase as well as late inflammatory phase. This suggested that opioid system might be involved in the centrally activated antinociceptive activity by cardamonin.

### 2.3. Toxicity Assessment

After seven days of observation, no mortality was reported at all doses of cardamonin used. During the observation period, the animals did not show any signs of abnormal behavior and locomotor activity. The observations were done qualitatively.

### 2.4. Motor Coordination Evaluation

Animals subjected to rota rod task after administration of cardamonin (10 mg/kg; i.p.) did not show any disturbance in motor coordination. The mean ± S.E.M. in the rota rod test for control, cardamonin (10 mg/kg) and diazepam (4 mg/kg) were 114.7 ± 5.33 s, 120.0 ± 0.00 s, and 38.83 ± 4.38 s, respectively.

## 3. Discussion

According to the Third National Health and Morbidity Survey 2006, 7% of the Malaysian population were suffering from chronic persistent pain [21]. Chronic pain has become a prevalent health problem among the older generation in Malaysia [22]. This has led to higher hospitalization rates among the elderly and severely interferes with the quality of life.

Non-steroidal anti-inflammatory drugs (NSAIDs), opioids, and analgesic adjuvants are the major classes of pharmacological therapies for pain relief currently available in modern medicine [23]. In spite of pain relieving effects, prolonged usage of these drugs leads to undesirable side effects such as gastrointestinal bleeding, renal toxicity, hypotension and respiratory depression [24,25]. These adverse effects have limited the usage of well-known effective pain relieving medicines. Research has been continuously carried out to seek for alternative pharmacologically potent analgesic treatment with fewer or milder side effects.

Previous studies have demonstrated an increase in NOS and TNF-α gene expression in the injected paw of animals after intraplantar administration of CFA [26]. It was postulated that NO capable of inducing peripheral hyperalgesia by regulating the expression of cyclooxygenase (COX) which then results in an increase of prostaglandin release [27]. Thus, with the reports of the inhibitory effect of cardamonin upon the release of pro-inflammatory mediators and also inhibition of both the NO and PGE_2_ [13,14,15], we suggest that cardamonin possess antinociceptive effect in both chemical- and thermal-induced nociception models in mice.

The present study demonstrated the antinociceptive effects of cardamonin, either through intraperitoneal or oral administration, at doses that did not produce impairment of motor coordination. It showed a dose-dependent inhibition in acetic acid-induced abdominal writhing test in mice. This result support the finding on antinociceptive activity of cardamonin in PBQ-induced writhing test [20]. The acetic acid–induced abdominal writhing model has been utilized as a screening tool for the assessment of antinociceptive and also anti-inflammatory properties of potential analgesic agents [28]. Following acetic acid administration into the peritoneal cavity of the animals, chemical mediators released directly or indirectly at the free nerve endings of sensory polymodal neurons. They also reported on the increase level of prostaglandins, especially PGE_2_ in the peritoneal fluid following acetic acid injection [29]. In line with the in vitro finding where cardamonin exerted inhibitory action of PGE_2_ secretion and down regulation of COX-2 gene expression, we suggest that the antinociceptive mechanism of cardamonin may be linked partly to the disruption of the LOX and/or COX in the peripheral tissues, leading to decrease in PGE_2_ synthesis, which then interferes with the mechanism of signal transduction in primary afferent nociceptors. It has also been previously reported that cardamonin inhibits inflammatory cytokines like TNF-α, IL-1β and IL-6 and interferes with the NF-κB signaling pathway [15] that may additionally explain the antinociceptive effects of cardamonin.

The commonly used analgesic agent NSAIDs inhibits the synthesis of prostaglandin, and in the present study, indomethacin exerted a significant inhibition in the acetic acid–induced pain model. As shown in Figure 2, cardamonin at the dose of 3 mg/kg has similar analgesic effect as indomethacin at dose 10 mg/kg. However, acetic acid–induced nociceptive model has good sensitivity but poor specificity. This is due to the suppressing effect of abdominal writhes by muscle relaxants or other types of drugs to misinterpretation of the results [30,31]. Thus, formalin-induced paw licking and hot plate test were carried out in the present study to avoid this problem.

The responses of formalin-induced paw licking is biphasic [32]. The first phase reflects the neurogenic pain whereby formalin-injected into the paw directly sensitize the C-fiber primary afferent nociceptors. The second phase is the result of inflammatory response caused by the action of inflammatory mediators, or to some extent, the central sensitization of the dorsal horn neurons [32,33]. Substance P and bradykinin was reported release during the neurogenic phase, whereas histamine, serotonin, prostaglandins and also bradykinin were involved in the inflammatory phase [34]. Centrally acting analgesics such as morphine inhibit nociception in both phases equally; in contrast, peripherally acting analgesics such as indomethacin only inhibit the second phase [33,34]. The present study indicates that cardamonin at all doses reduce paw lickings induced by formalin in both phases. Thus it was suggested that cardamonin works both centrally and peripherally, which also implies that cardamonin not only possess antinociceptive response but also anti-inflammatory activity.

In line with its centrally acting properties, cardamonin exert significant prolongation in response latency time to thermal stimuli as in the hot plate assessment. Hot plate test is a sensitive and specific thermal model for the evaluation of the involvement of central analgesic activity, or in other words supra-spinal activity [35,36]. Thus, the present study provides strong evidence that cardamonin exert centrally mediated antinociceptive activity as it decreases nociception in the first phase of formalin test and increased nociceptive threshold in the hot plate test. In addition, pre-treatment with non-selective opioid antagonist, naloxone has significantly antagonized the antinociceptive effect of cardamonin and morphine in both the formalin-induced paw licking and hot plate test. These finding suggest that the central antinociceptive mechanism of cardamonin could involve the activation of opioid receptors or modulation of the effect of endogeneous opioid peptides.

In order to further understand the antinociceptive action of cardamonin, capsaicin-induced neurogenic paw licking test was carried out. Capsaicin, the pungent ingredient of hot chili peppers, upon administered, it directly stimulates the transient receptor potential cation channel V1 (TRPV_1_) which found on the sensory C-fibers. Capsaicin also mediates the release of excitatory amino acids (glutamate and aspartate), nitric oxide and pro-inflammatory mediators and thus transmitting the nociceptive impulses to the central spinal system [37]. In this study, cardamonin also exhibit significant reduction in nociceptive responses in capsaicin-induced paw licking assessment.

Another interesting finding of this study reveals that cardamonin exhibit significant inhibitory nociceptive response against intraplantar injection of glutamate into mouse hind paw. Glutamate-induced nociceptive responses appears to involve peripheral, spinal and supra-spinal sites of action and is mediated by both of the activation of *N*-methyl-*D*-aspartate (NMDA) and α-amino-3-hydroxyl-5-methyl-isoxazolepropionate (non-NMDA) receptors, as well as by nitric oxide release or by some NO-derived substances. The release of nitric oxide eventually increased the synthesis or release of pro-inflammatory mediators such as cytokine, reactive oxygen species (ROS) as well as prostanoids followed by enhanced inflammatory reaction [38]. The present study strongly suggests that antinociceptive activity induced by cardamonin in the glutamate test, at least in part, could be due to its interaction with the glutamatergic system or its ability to inhibit NO production.

Finally, the present study provides convincing evidence that systemic administration of cardamonin was largely devoid of significant effect on motor coordination of animals in the rota rod assessment. Therefore, the possibility of non-specific muscle relaxation and sedative effects of cardamonin-induced antinociception could be eliminated. The result of preliminary acute toxicity investigation showed that no occurrence of animal mortality over the period of observation. The study conducted qualitatively by observing signs of toxicity indicated that cardamonin have a reasonably low toxicity profile and should be regarded as safe while detailed quantitative assessments to be carried out in the future.

## 4. Materials and Methods

### 4.1. Plant Material

Five kilograms of *Boesenbergia rotunda* were purchased from the local market in Serdang, Malaysia and was authenticated by a resident botanist at the Institute of Bioscience, Universiti Putra Malaysia (IBS, UPM), Malaysia. A voucher specimen (SK1780/10) was deposited at the Herbarium, Laboratory of Natural Products, IBS, UPM and small part of the rhizomes were cultivated at the Medicinal Plant Garden, IBS, UPM for future reference.

### 4.2. Extraction and Isolation

**Figure 6:**
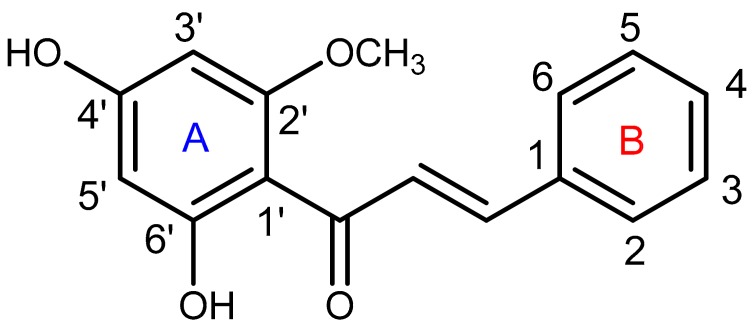
The chemical Structure of cardamonin.

The fresh rhizomes of *B*. *rotunda* were sliced into small flat pieces and dried under shadow for one weak. The dried rhizomes were ground into fine powder by using domestic food processor. The dried powder 2.5 kg was dissolved in distilled methanol for two-three days. The methanolic extract was filtrate and concentrate on rotary evaporator to obtained 255 gm crude extract. The methanolic extract was subjected to solvent extraction. The crude methanolic extract was dissolved in 250 mL distilled water and transferred into separating funnel. About 150 mL hexane was added into aqueous layer and subsequently extracted with chloroform, ethyl acetate, and butanol. The chloroform layer finally passed over sodium sulfate anhydrous to remove the moisture. The chloroform extract was subjected to flash column chromatography by using ethyl acetate and hexane as eluents. Finally, the compound (Figure 6) was purified from chloroform extract and identify as cardamonin (CARD) after performing the detailed NMR spectroscopic and chromatographic methods, respectively. The purity of the compound was 98.0%.

*(Cardamonin): (E)-1-(4′,6′dihydroxy-2′-methoxyphenyl)-3-phenylprop-2-en-1-one*. (*E*)-1-(4′,6′-dihydroxy-2′-methoxyphenyl)-3-phenylprop-2-en-1-one: yellow needles crystals: m.p. 192–194 °C, EI-MS *m*/*z* 270.23, (molecular formula C_16_H_16_O_4_). IR max (cm^−1^, KBr disc): 3256 (OH), 1632 (C=O), 1544, 1490, 1288, 1326, 1228 cm^−1^. ^1^HNMR (CDCl_3_, 500 MHz): *δ* 7.92 (d, 1H, *J* = 15.5 Hz, Hβ), 7.80 (d, 1H, *J* = 15.5 Hz, Hα), 7.62 (br, d, 2H, H-2,6), 7.46 (m, 3H, H-3,4,5), 6.12 (br, s, 1H, H-3′), 5.98 (br, s, 1H, H-5′), 3.85 (s, 3H, OMe, C-2′). int.): *m*/*z* 270 ([M^+^], 269 (54), 253 (5), 193 (100), 131 (35, 103 (39), 77 (32).

### 4.3. Experimental Animals

All animal care and the antinociceptive experimental procedures performed were in accordance with the ethical guidelines for investigations of experimental pain in conscious animals (Zimmermann, 1983) and approved by the Animal Care Unit Committee (ACUC), Faculty of Medicine and Health Sciences, Universiti Putra Malaysia (UPM/FPSK/PADS/BR-UUH/00425). The antinociceptive experiments were carried out using male ICR mice (20–30 g). They were housed in groups of 10 per cage and maintained on a 12/12-h light/dark cycle (lights were switched on at 06:00 h), at the animal house facility, Faculty of Medicine and Health Sciences, Universiti Putra Malaysia. Food and water were available ad libitum, except during the experimental procedure. The animals were habituated to the condition of the laboratory at least 2 h before testing and were used only once throughout the experiments. The number of animals and intensities of noxious stimuli used were the minimum necessary to demonstrate consistent effects of drug treatments. All efforts were made to minimize animal suffering. At the end of the experiments, the animals were anesthetized and euthanized by cervical dislocation. In all experiments, data were collected by a blinded, randomized, and controlled design.

### 4.4. Drugs and Chemicals

Tween 20, absolute ethanol, acetic acid, formalin, morphine hydrochloride, naloxone, indomethacin, diazepam, capsaicin, capsazepine, and glutamate were purchased from Sigma Chemical Co. (St. Louis, MO, USA). All drugs used were dissolved in physiological saline (0.9% NaCl). Cardamonin and indomethacin were dissolved in ethanol, Tween 20 and distilled water in 5:5:90 (*v*/*v*/*v*) fractions. Respective controls received only solvent vehicle, whereby it had no effect per se on nociceptive responses. All drugs, chemicals, and CARD solutions were freshly prepared and administered intraperitoneally (i.p.) in a volume of 10 mL/kg, unless otherwise stated in the method.

### 4.5. Evaluation of the Antinociceptive Activity

#### 4.5.1. Acetic Acid–Induced Abdominal Writhing Test

The acetic acid–induced abdominal writhing test was conducted as previously described [39]. Mice were pre-treated with CARD (0.3, 1, 3 and 10 mg/kg; i.p.), 30 min prior to injection of 0.6% acetic acid (10 mL/kg; i.p.). Indomethacin (10 mg/kg) was used as reference drug and was administered by i.p. route, 30 min before the nociceptive agent. Following the injection of acetic acid, the animals were immediately placed into a perspex chamber, and the number of writhing was recorded for 30 min, starting from 5 min post-injection. Antinociceptive activity of CARD was expressed as a reduction in the mean number of abdominal writhes in the group pre-treated with CARD compared with the control group.

#### 4.5.2. Formalin-Induced Paw Licking Test

The formalin-induced paw licking test was carried out as previously described [40]. Mice used were individually adapted in an observation chamber made of transparent acrylic. CARD (0.3, 1, 3, and 10 mg/kg; i.p.) or vehicle (10 mL/kg; i.p.) was administered 30 min before the formalin injection. Indomethacin (10 mg/kg; i.p.) and morphine (5 mg/kg; s.c.) were used as positive control drug and were administered 30 min and 1 h before the test. After 30 min, 20 μL of 2.5% of formalin solution (*v*/*v* in distilled water) was injected subcutaneously into the ventral surface of the right hind paw of the mice. The amount of time (in seconds) spent on licking and biting the injected paw was recorded up to 35 min after formalin injection as an indicator of nociceptive behavior. The initial nociceptive scores normally peaked at 0–5 min (early phase) and 15–35 min (late phase) after formalin injection, representing the neurogenic and inflammatory pain responses, respectively.

#### 4.5.3. Hot Plate Test

The antinociceptive property of cardamonin for thermal noxious stimuli was evaluated according to the method described previously [41]. The metal surface of the hot plate (Model 7280, Ugo Basile, VA, Italy) was set and maintained at temperature of 52 ± 0.2 °C. Mice were then placed into the Plexiglas cylinder on the heated metal surface individually and the time elapsed between placement and licking the forepaws, or jumping were recorded as response latency time. The reaction time was recorded before and at 30, 60, 90, 120, 180 and 210 min after administration of cardamonin (0.3, 1, 3, and 10 mg/kg; i.p.) or vehicle (10 mL/kg; i.p.). Morphine (5 mg/kg; s.c.) was used as a reference drug. A cut-off time of 20 s was defined as complete analgesia and to avoid tissue damage.

### 4.6. Investigation of the Mechanisms of Action

#### 4.6.1. Involvement of the TRPV_1_ Receptor

The involvement of TRPV_1_ in antinociceptive activity by CARD was investigated by using capsaicin-induced paw licking model. The procedure implemented was similar to that described previously [42]. Mice were pre-treated with CARD (0.3, 1, 3, and 10 mg/kg; i.p.), capsazepine (0.17 mmol/kg; i.p.), indomethacin (10 mg/kg; i.p.) or vehicle (10 mL/kg; i.p.) prior to injection of 20 μL of capsaicin (1.6 μg/paw) intraplantarly (i.pl) into the ventral surface of the right hind paw of the mice. The animals were observed individually for 5 min after capsaicin injection. The amount of time spent licking the injected paw was recorded and was considered as an indication of pain behavior.

#### 4.6.2. Involvement of the Glutamate Receptor

The participation of glutamate receptor in antinociceptive activity by cardamonin was investigated through glutamate-induced paw licking model. The experiment was carried out as previously described [38]. Mice were pre-treated with CARD (0.3, 1, 3, and 10 mg/kg; i.p.), indomethacin (10 mg/kg; i.p.) or vehicle (10 mL/kg; i.p.). After 30 min, 20 μL of glutamate (10 μmol/paw; i.pl.) was injected into the ventral surface of right hind paw of the mice. The mice were then observed individually for 15 min after glutamate injection. The amount of time spent licking the glutamate-injected paw was recorded and was considered as an indication of pain behavior.

#### 4.6.3. Involvement of the Opioid Receptors

To investigate the possible involvement of the opioid system in the antinociceptive activity of cardamonin, separate groups of mice were pre-treated with the non-selective opioid receptor antagonist, naloxone (5 mg/kg; i.p.) 15 min prior to the administration of cardamonin (1 mg/kg; i.p.), morphine (5 mg/kg; s.c.) or vehicle (10 mL/kg; i.p.). After 30 min (for i.p. administration) and 60 min (for s.c. administration), the mice were subjected to the formalin-induced paw licking test and hot plate test [42].

### 4.7. Toxicity Assessment

The acute toxicity study was conducted to assess the toxicity of cardamonin as described previously [41]. Animals were fasted overnight prior to the test with free access to water ad libitum. Mice were divided into groups and were administered with experimental doses of cardamonin (0.3, 1, 3 and 10 mg/kg) orally (p.o.) and intraperitoneally (i.p.). The control group received the vehicle (10 mL/kg). After administration of cardamonin, the animals were observed for 4 h for any abnormal behaviors, respiratory distress, motor impairment, sedation, and hyper-excitability qualitatively. In addition, any incidence of mortality was recorded up to 24 h after administration of cardamonin. Animals were given free access to standard pellet and water throughout the study.

### 4.8. Motor Coordination Test

Rota-rod test was conducted to investigate the possible sedative and motor-coordination effect of cardamonin. The method employed was similar to that described previously with slight modification [43]. Mice that successfully remain on the revolving bar of the rota rod apparatus (Model 7600, Ugo Basile) revolving at a speed of 20 rounds per min for two consecutive periods of 60 s were selected 24 h prior to the test. The selected mice were administered with cardamonin (10 mg/kg; i.p.), vehicle (10 mL/kg; i.p.) or diazepam (4 mg/kg; i.p.) 30 min prior to the experiment. Motor performance was evaluated as the latency of permanence(s) on the revolving bar up to 120 s at the time of 30, 60, and 90 s after the treatment. The average time of the animals remain on the revolving bar was recorded.

### 4.9. Statistical Analysis

The data collected was expressed as mean ± S.E.M. obtained from 6 animals per group and analyzed using one-way ANOVA followed by Dunnett’s post hoc test, unless otherwise stated. The differences between means were considered as statistically significant at *p* < 0.05. The experimental ED_50_ (effective dose producing a 50% reduction in abdominal writhes) and its 95% confidence intervals (CI) were determined by linear regression using GraphPad Prism (Version 5, GraphPad Software, La Jolla, CA, USA). The percentages of inhibition were calculated by comparing the results of treatment group with control group.

## 5. Conclusions

In conclusion, the present study demonstrated that systemic administration of cardamonin at a dose which does not cause any toxic effects and interference of motor co-ordination exerted significant peripheral and central antinociception when assessed in the chemical- and thermal-induced nociception test models in mice through the involvement of TRPV_1_, glutamate, and opioid receptors. The precise mechanism underlying the antinociceptive activity of cardamonin remains to be investigated. Currently, a study to determine the possible mechanism(s) of action responsible for cardamonin-induced antinociceptive activity is in progress.

## Figures and Tables

**Table 1:** Effect of cardamonin on the hot plate test in mice. Results were expressed in mean ± S.E.M of latency time (s) of 6 mice. Statistical significance was determined by two-way ANOVA followed by Bonferroni post hoc test.

Treatment	Dose	Latency Time (s)							
	(mg/kg)	0 min	30 min	60 min	90 min	120 min	150 min	180 min	210 min
**Control**		6.17 ± 0.17	6.83 ± 0.31	6.67 ± 0.33	6.67 ± 0.21	6.83 ± 0.31	6.67 ± 0.21	6.83 ± 0.31	6.33 ± 0.21
**Cardamonin (i.p.)**	0.3	6.34 ± 0.16	6.99 ± 0.52	7.01 ± 0.27	8.26 ± 0.33	8.28 ± 0.56	9.08 ± 1.09 *	8.36 ± 0.59	6.83 ± 0.55
	1	7.05 ± 0.22	6.84 ± 0.56	7.03 ± 0.16	7.11 ± 0.28	8.04 ± 0.67	8.97 ± 0.44 *	8.50 ± 0.37	7.22 ± 0.26
	3	6.95 ± 0.25	6.83 ± 0.25	7.59 ± 0.25	7.65 ± 0.16	8.37 ± 0.41	8.92 ± 0.24 *	7.52 ± 0.51	6.71 ± 0.36
	10	6.63 ± 0.20	7.45 ± 0.46	8.08 ± 0.46	9.14 ± 0.72 *	10.09 ± 0.89 ***	9.21 ± 0.83 **	8.07 ± 0.33	7.48 ± 0.15
**Naloxone (i.p.) + Cardamonin (i.p.)**	1 + 5	7.30 ± 0.24	8.70 ± 0.28	8.54 ± 0.32	8.60 ± 0.25	11.33 ± 0.59 ^###^	11.62 ± 0.50 ^##^	9.40 ± 0.58	8.45 ± 0.66
**Morphine (s.c.)**	5	7.50 ± 0.34	18.33 ± 0.67 ***	17.00 ± 0.82 ***	16.33 ± 0.42 ***	16.33 ± 0.99 ***	15.33 ± 0.62 ***	15.17 ± 0.40 ***	14.83 ± 0.40 ***
**Naloxone (i.p.) + Morphine (s.c.)**	5 + 5	7.30 ± 0.29	8.93 ± 0.82 ^###^	11.18 ± 0.74 ^###^	10.76 ± 0.61 ^###^	10.10 ± 0.84 ^###^	9.01 ± 0.83 ^###^	7.64 ± 0.30 ^###^	6.92 ± 0.30 ^###^

* *p* < 0.05 as compared to control; ** *p* < 0.01 as compared to control; *** *p* < 0.001 as compared to control; ^##^ *p* < 0.01 as compared to the group receiving appropriate drug/compound at the same dose without naloxone; ^###^ *p* < 0.001 as compared to the group receiving appropriate drug/compound at the same dose without naloxone.
